# Evaluation of Clinical Safety and Performance of the Pipit-NC Percutaneous Transluminal Coronary Angioplasty (PTCA) Balloon Dilatation Catheter in Coronary Artery Stenosis: A Post-market Clinical Study

**DOI:** 10.7759/cureus.95776

**Published:** 2025-10-30

**Authors:** Atul D Abhyankar, Vikas Patel, Maulik Prajapati

**Affiliations:** 1 Cardiology, Shri B. D. Mehta Mahavir Heart Institute, Surat, IND

**Keywords:** balloons, calcification, coronary artery disease, coronary stenosis, percutaneous coronary intervention

## Abstract

Aim: The present study aimed to evaluate the clinical safety and performance of the Pipit-NC PTCA balloon dilatation catheter (Sahajanand Medical Technologies Limited, Surat, India) in real-world PCI procedures for the treatment of coronary artery stenosis.

Materials and Methods: This was a prospective, observational, single-center, single-arm, investigator-initiated, post-market clinical study that enrolled 87 patients who were treated with at least one Pipit-NC PTCA balloon dilatation catheter during their index PCI procedure. The primary endpoints were device success and procedural success. Secondary endpoints included procedural complications and in-hospital safety outcomes, such as all-cause mortality, any myocardial infarction, and any revascularization.

Results: A total of 134 Pipit-NC PTCA balloon catheters were utilized during the index procedures in 87 patients (mean age 60.68 ± 10.99 years; 79.3% male) with 90 coronary lesions, including 44 balloons for pre-dilation and 90 for post-dilatation. The treated lesion included 47 (52.2%) calcified lesions, 16 (17.8%) chronic total occlusions, and 10 (11.1%) lesions in tortuous vessels. The balloon demonstrated consistent performance during both pre-dilatation (mean pressure: 16.86 ± 1.85 atm) and post-dilatation (mean pressure: 25.93 ± 2.97 atm). The Pipit-NC balloon achieved 100% device success - meeting all criteria for safe and effective delivery, function, and retrieval - and 100% procedural success, with no in-hospital target lesion failure events observed.

Conclusion: The Pipit-NC PTCA balloon dilatation catheter demonstrated excellent clinical safety and performance in real-world clinical practice, achieving 100% device and procedural success without complications, even in complex coronary lesions. Its effectiveness in both pre- and post-dilatation applications supports its utility in index PCI procedures.

## Introduction

Coronary artery disease remains a leading global cause of mortality, but percutaneous coronary intervention (PCI) has transformed its management, with over 2 million procedures performed annually [1]. Advances in PCI techniques have improved patient outcomes by reducing procedural complications and enhancing long-term success [2]. Yet, stent delivery complications persist in 2.7-3.3% of cases, with balloon expansion potentially causing vessel wall injury and coronary dissection [3]. These dissections vary in severity, which can lead to myocardial infarction or life-threatening complications.

Non-compliant (NC) balloons are vital tools in modern PCI due to their precise, high-pressure dilation capability. Unlike semi-compliant balloons, these devices have minimal elasticity, ensuring uniform radial force along the vessel wall, regardless of inflation pressure. This property enhances stent deployment accuracy and reduces complications, such as side branch dissection in complex bifurcation lesions [4]. Additionally, optical coherence tomography (OCT) studies confirm that these balloons achieve greater luminal gain than semi-compliant alternatives, highlighting their efficacy across diverse anatomies. Beyond bifurcations, NC balloons excel in various lesion types, including calcified lesions, chronic total occlusions, and tortuous vessels, as their consistent expansion during pre-dilation ensures effective lesion preparation [5]. Among these challenging lesion subsets, calcified coronary lesions remain particularly difficult to treat due to their rigidity and resistance to balloon expansion, often leading to suboptimal stent deployment and increased complication risk.

Despite these advancements, real-world data on the performance of specific NC balloon catheters in diverse, unselected patient populations remain limited. Most studies focus on controlled settings or specific lesion types, leaving a gap in understanding how these devices perform under routine clinical conditions across varied anatomical and patient characteristics.

This study aims to address this gap by evaluating the clinical safety and performance of the Pipit-NC percutaneous transluminal coronary angioplasty (PTCA) balloon dilation catheter (Sahajanand Medical Technologies Limited, Surat, India) in real-world PCI procedures for the treatment of coronary artery stenosis.

## Materials and methods

Study design and patient population

This was a prospective, observational, single-center, single-arm, investigator-initiated post-market clinical study conducted between July 2021 and September 2024 at a tertiary care centre in India. This study was registered with the Clinical Trials Registry - India (CTRI) (Registration no.: CTRI/2023/08/056616).

A total of 87 patients above the age of 18 years who were treated with at least one Pipit-NC PTCA balloon dilatation catheter during the index PCI procedure (pre-dilation before stent deployment and/or post-dilatation after stent deployment) were enrolled in the study. The study protocol was approved by the Institutional Ethics Committee. The study design and procedures conformed to the principles of the Declaration of Helsinki and the International Council for Harmonisation: Good Clinical Practice (ICH-GCP) guidelines. Written informed consent was obtained from all participants prior to the index procedure, including consent for study participation and the use of anonymized clinical data for research and publication purposes.

Study device

The Pipit-NC PTCA balloon features a tri-fold design and is available in sizes ranging from 2.00 mm to 5.00 mm in diameter and 6 mm to 30 mm in length. The Pipit-NC PTCA dilatation catheter is designed for easy exchange of the catheter using a standard 0.014-inch PTCA guidewire. The catheter comprises a dual-lumen shaft: the proximal shaft consists of a PTFE-coated stainless-steel tube bonded to a female luer connector, while the distal shaft features a nylon outer layer and an inner lumen, with a balloon affixed by thermal bonding at both ends. To improve trackability and navigation through tortuous coronary anatomy, the distal shaft is coated with a biocompatible hydrophilic coating. The guidewire enters the catheter's tip and advances coaxially out of the distal Rx port, thereby allowing both coaxial guidance and rapid exchange of the catheter with a single standard-length guidewire. Two marked sections located on the proximal shaft, at distances from the tip, indicate catheter position relative to the tip of either a brachial (900 mm) or femoral (1000 mm) guiding catheter.

Study endpoints and definitions

The primary endpoints of this study were device success and procedural success. Device success was defined as the successful delivery of the balloon catheter to the target lesion, followed by the successful inflation, deflation, and retrieval of the device into the guiding catheter. Device success also required the absence of procedure-related complications, including coronary perforation, dissection, clinically significant arrhythmias requiring intervention, reduction in coronary blood flow, or balloon rupture at or below the rated burst pressure. Procedural success was defined as device success without target lesion failure. Target lesion failure was defined as a composite of cardiovascular death, target vessel myocardial infarction (TV-MI), or clinically driven target lesion revascularization (CD-TLR) during hospitalization. Secondary endpoints included individual procedural parameters and safety endpoints. Procedural parameters, evaluated from the start to the end of the PTCA procedure, comprised the incidence of coronary perforation, dissection, thrombosis, and clinically significant arrhythmias necessitating therapeutic intervention. The safety endpoints were assessed until hospital discharge, which included all-cause mortality, any myocardial infarction, and any repeat revascularization procedure.

Data collection

Data were collected for all enrolled patients from the time of the index procedure through hospital discharge. Baseline demographic characteristics, clinical history, lesion and procedural characteristics, and device-specific parameters were documented using standardized case report forms (CRFs). Procedural details, including balloon delivery, inflation, and deflation times, and retrieval success, were recorded intraoperatively. Adverse events such as coronary perforation, dissection, thrombosis, arrhythmias, and balloon rupture were captured in real-time during the procedure. Post-procedural events, including myocardial infarction, target lesion revascularization, and mortality, were monitored and recorded throughout the index hospitalization.

Statistical analysis

Categorical variables were analyzed and expressed as numbers and percentages. Continuous variables were expressed as mean ± SD. Statistical analyses were performed using the Statistical Package for Social Sciences version 21.0 (SPSS IBM Corp., Armonk, NY).

## Results

Patient demographics and baseline characteristics

A total of 87 patients were enrolled in the study. The mean age was 60.68 ± 10.99 years, with a predominance of males (n=69; 79.3%). Hypertension was present in 34 patients (39.1%), diabetes mellitus in 21 patients (24.1%), and hypercholesterolemia in 47 patients (54.0%). The clinical presentation of ST-elevation myocardial infarction (STEMI) was observed in 50 patients (57.5%). Multivessel disease was observed in six (6.8%) patients. Baseline demographics and clinical characteristics of patients are summarized in Table 1.

**Table 1:** Baseline and demographic characteristics of patients treated with the Pipit-NC PTCA balloon dilatation catheter Data are presented as mean ± SD or n (%). CABG: Coronary artery bypass grafting; CAD: Coronary artery disease; MI: Myocardial infarction; PCI: Percutaneous coronary intervention

Baseline Characteristics	Values (total = 87 patients)
Age, years	60.68 ± 10.99
Male	69 (79.3)
Heart rate, beats per minute	82.10 ± 6.55
Systolic blood pressure, mmHg	132.46 ± 8.63
Diastolic blood pressure, mmHg	76.63 ± 5.06
Medical history	
Hypertension	34 (39.1)
Diabetes mellitus	21 (24.1)
Hypercholesterolemia	47 (54.0)
Current smoker	14 (16.1)
Family history of CAD	12 (13.8)
Previous myocardial infarction	4 (4.6)
Previous PCI	3 (3.4)
Previous CABG	1 (1.1)
Anginal status	
ST-elevation MI	50 (57.5)
Unstable angina	30 (34.5)
Non-ST elevation MI	3 (3.4)
Stable angina	4 (4.6)
Antiplatelet therapy given pre-procedure	
Clopidogrel	1 (1.1)
Ticagrelor	86 (98.9)
Single-vessel disease	81 (93.1)
Double-vessel disease	5 (5.7)
Triple-vessel disease	1 (1.1)

Lesion and procedural characteristics

A total of 90 lesions were treated, with lesion characteristics detailed in Table 2. Lesion complexity included 47 (52.2%) calcified lesions, 16 (17.8%) of chronic total occlusions, 10 (11.1%) lesions in tortuous vessels, and 2 (2.2%) of bifurcation lesions.

**Table 2:** Lesion details and characteristics of patients who underwent PTCA Data are presented as mean ± SD or n (%).

Lesion Details	Values (total = 90 lesions)
Target vessels	
Left anterior descending artery	36 (40.0)
Left circumflex artery	22 (24.4)
Right coronary artery	28 (31.1)
Left main coronary artery	2 (2.2)
Ramus intermedius	2 (2.2)
Indications	
De novo	89 (98.9)
In-stent restenosis	1 (1.1)
Lesions characteristics	
Calcified	47 (52.2)
Chronic total occlusion	16 (17.8)
Tortuosity	10 (11.1)
Bifurcation	2 (2.2)
Percentage diameter stenosis	91.78 ± 5.97

Procedural details are presented in Table 3. A total of 91 drug-eluting stents (DES) were implanted, of which 74 (81.3%) were everolimus-eluting and 17 (18.7%) were sirolimus-eluting. The Pipit-NC PTCA balloon dilatation catheter was used in 134 balloon deployments (44 pre-dilatation, 90 post-dilatation). Pre-dilatation balloons had a mean length of 8.48 ± 2.04 mm, diameter of 2.81 ± 0.56 mm, and maximum inflation pressure of 16.86 ± 1.85 atm. Post-dilatation balloons had a mean length of 8.30 ± 1.97 mm, diameter of 2.96 ± 0.63 mm, and maximum inflation pressure of 25.93 ± 2.97 atm.

**Table 3:** Stent and balloon specifications used in coronary intervention procedures Data are presented as mean ± SD or n (%). DES: Drug-eluting stent

Stent and balloon specification	Values (total = 87 patients)
Stent details	
Total stents, n	91
Stent length, mm	24.24 ± 8.98
Stent diameter, mm	3.02 ± 0.52
DES, n	91 (100)
Everolimus-eluting stent	74 (81.3)
Sirolimus-eluting stent	17 (18.7)
Balloon details	
Total balloons, n	134
Pre-dilatation balloons, n (%)	44 (32.8)
Balloon length, mm	8.48 ± 2.04
Balloon diameter, mm	2.81 ± 0.56
Maximum inflation pressure, atm	16.86 ± 1.85
Maximum inflation time, sec	30.00 ± 0.00
Post-dilatation balloon, n(%)	90 (67.2)
Balloon length, mm	8.30 ± 1.97
Balloon diameter, mm	2.96 ± 0.63
Maximum inflation pressure, atm	25.93 ± 2.97
Maximum inflation time, sec	30.23 ± 1.70
Balloon length	
6 mm	33 (24.6)
8 mm	63 (47.0)
10 mm	22 (16.4)
12 mm	14 (10.5)
15 mm	2 (1.5)
Balloon diameter	
2 mm	14 (10.5)
2.25 mm	13 (9.7)
2.5 mm	23 (17.2)
2.75 mm	16 (11.9)
3 mm	30 (22.4)
3.25 mm	3 (2.2)
3.5 mm	21 (15.7)
4 mm	11 (8.2)
4.5 mm	3 (2.2)
Device success	134 (100)
Procedure success	87 (100)

Primary and secondary endpoints

Device success was achieved in 100% of 134 deployments. Procedural success was achieved in 100% of cases. No procedural complications, including coronary perforation, dissection, thrombosis, or arrhythmias requiring intervention, were reported. No instances of all-cause mortality, cardiovascular death, myocardial infarction, or any revascularization were observed during hospitalization.

## Discussion

The findings of this study highlighted the excellent clinical performance and safety of the Pipit-NC PTCA balloon dilatation catheter in complex coronary interventions. The study included patients with complex lesion characteristics, including 52.2% calcified lesions, 17.8% chronic total occlusions, and 11.1% lesions in tortuous vessels. Despite these complexities, the study achieved a 100% procedural success and device success across 134 balloon deployments, with no procedural complications such as coronary perforation, dissection, or thrombosis. And no in-hospital safety outcomes were observed, such as all-cause mortality, any myocardial infarction, or any revascularization.

These findings are consistent with a previous study that evaluated the River NC® coronary balloon catheter (Balton, Poland) in 42 patients, reporting a 100% device success rate. Notably, 73.8% of the patients had multivessel disease, with a high prevalence of calcified lesions. [6]. Similarly, a study performed by Pradhan et al. [7] assessed the clinical performance of the Mozec™ NC Rx PTCA balloon dilatation catheter (Meril Life Sciences Pvt. Ltd., Vapi, India) and reported 100% procedural and device success. However, the study included relatively simple lesions compared to the present study, i.e., only 18% of lesions were calcified. The present study, however, stands out due to the higher prevalence of calcified lesions, further emphasizing the Pipit-NC PTCA balloon dilatation catheter’s ability to perform well in more difficult anatomical scenarios. Data from the COBIS II registry show that NC balloons improve procedural success and are associated with lower MACE during long-term follow-up, supporting the clinical benefits observed in our study [4].

A case was reported by Martins et al. [8] in which coronary perforation occurred after pre-dilatation with an NC Trek™ balloon catheter (Abbott Vascular, Temecula, CA). In contrast, the Pipit-NC PTCA balloon dilatation catheter demonstrated effective utility during both pre-dilatation and post-dilatation in the current study, highlighting its versatility in clinical practice. Of the 134 total balloon deployments, 44 were used for pre-dilatation of the lesion prior to stent placement, achieving adequate lesion preparation without complications. This dual applicability of the Pipit-NC PTCA balloon dilatation catheter may reduce the need for multiple devices and improve procedural efficiency. Furthermore, the balloon consistently tolerated high pressures during post-dilatation (mean 25.9 atm), aligning with or exceeding the inflation parameters reported for other leading NC balloons such as NC Trek and Sapphire II (OrbusNeich, Fort Lauderdale, FL). The ability to safely deliver both moderate and high-pressure inflations without procedural complications reinforces the mechanical reliability of the Pipit-NC PTCA balloon dilatation catheter and supports its role in complex coronary interventions requiring precision and durability.

Managing calcified coronary lesions during the index procedure remains a challenge due to their association with increased procedural complexity and complication rates. Calcified plaques could impede optimal balloon expansion and stent deployment, leading to risks such as dissection, perforation, and stent under-expansion [9-11]. Recent studies have underscored these challenges, such as a study by Suzuki et al. [12], which reported that patients with severely calcified lesions had a higher incidence of device-oriented composite endpoints (DoCE), including coronary dissection and perforation, compared to those without such calcifications. Similarly, a review article highlighted that calcified lesions are associated with lower procedural success and an increased risk of both early and late complications [9]. The Pipit-NC PTCA balloon dilatation catheter offers superior technical features suited for complex PCI. Bench tests show excellent shape memory and consistent high-pressure performance. Its hydrophilic coating enhances trackability, while the uncoated inflation zone prevents slippage, improving stability in calcified lesions. These design elements support their high success rates in this study.

Moreover, the patient profile in this study reflects real-world PCI practice, with a predominance of middle-aged males and common comorbidities like hypertension, diabetes, and hypercholesterolemia. The high rate of acute coronary syndromes underscores the device’s effectiveness in urgent, high-risk cases. The excellent procedural outcomes in this representative cohort support the Pipit-NC balloon’s broad clinical applicability.

Limitations

This study was conducted at a single center with a relatively small sample size. Additionally, it focused on short-term in-hospital outcomes. The lack of comparative analysis with other balloon catheters and potential operator variability may also influence the findings. Larger multi-center studies are needed to confirm these results.

## Conclusions

The Pipit-NC PTCA balloon dilatation catheter demonstrated optimal safety and clinical performance in routine clinical use. With 100% device and procedural success and no adverse in-hospital outcomes, the balloon catheter demonstrated effectiveness for both pre- and post-dilation, even in patients with complex lesion characteristics. 
